# Nanocluster Aerosols from Ozone–Human Chemistry Are Dominated by Squalene–Ozone Reactions

**DOI:** 10.1021/acs.estlett.4c00289

**Published:** 2024-06-21

**Authors:** Shen Yang, Dusan Licina

**Affiliations:** Human-Oriented Built Environment Lab, School of Architecture, Civil and Environmental Engineering, École Polytechnique Fédérale de Lausanne (EPFL), 1015 Lausanne, Switzerland

**Keywords:** ozone chemistry, ammonia, human skin lipids, particle formation, fatty acid

## Abstract

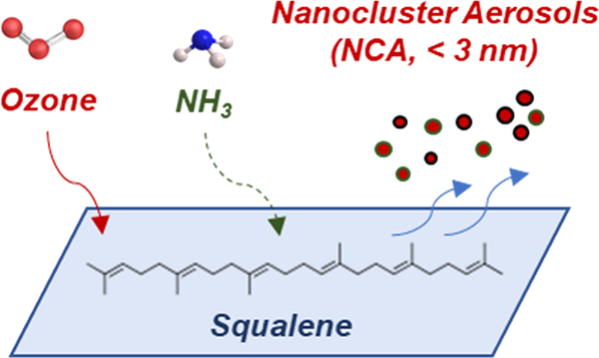

Nanocluster aerosols (NCAs, <3 nm particles) are associated with climate feedbacks and potentially with human health. Our recent study revealed NCA formation owing to the reaction of ozone with human surfaces. However, the underlying mechanisms driving NCA emissions remain unexplored. Squalene is the most abundant compound in human skin lipids that reacts with ozone, followed by unsaturated fatty acids. This study aims to examine the contribution of the squalene–ozone reaction to NCA formation and the influence of ozone and ammonia (NH_3_) levels. In a climate-controlled chamber, we painted squalene and 6-hexadecenoic acid (C16:1n6) on glass plates to facilitate their reactions with ozone. The squalene–ozone reaction was further investigated at different ozone levels (15 and 90 ppb) and NH_3_ levels (0 and 375 ppb). The results demonstrate that the ozonolysis of human skin lipid compounds contributes to NCA formation. With a typical squalene-C16:1n6 ratio found in human skin lipids (4:1), squalene generated 40 times more NCAs than did C16:1n6 and, thus, dominated NCA formation. More NCAs were generated with increased ozone levels, whereas increased NH_3_ levels were associated with the stronger generation of larger NCAs but fewer of the smallest ones. This study experimentally confirms that NCAs are primarily formed from squalene–ozone reactions in ozone–human chemistry.

## Introduction

Nanocluster aerosol (NCA) particles represent airborne nanoparticles that are <3 nm in diameter. Originating from traffic emissions^1^ and atmospheric processes,^2,3^ NCAs constitute a considerable fraction of outdoor aerosols by number^4,5^ and could become climatically relevant aerosol particles outdoors, depending on their physiochemical properties and atmospheric conditions.^6,7^ Despite the unknown health effects of exposure to NCAs, concerns have been raised because of their ability to deeply penetrate the human lungs and even reach the brain.^8,9^ In indoor environments where humans spend most of their time,^10^ NCAs could dominate the total particle number.^11^ Indoor NCAs could be transported from outdoors and generated from high-temperature processes (e.g., cooking and burning candles),^12,13^ heated surfaces,^14,15^ and ozone–terpene reactions.^16^ Additionally, ozone–human chemistry represents an intriguing yet understudied source of indoor NCAs. Our previous studies were the first to report NCA formation via ozone–human chemistry^17^ and ozone chemistry on worn clothing.^18^ Moreover, our recent study showed that the NCAs formed during ozone–human chemistry can grow into ultrafine particles ranging from 10 to 55 nm, depending on ventilation and indoor air movement.^19^ Nevertheless, the driving mechanisms of ozone–human chemistry generating NCAs remain unexplored. A fundamental question yet to be addressed is which constituent of human skin lipids plays the most significant role in NCA formation in the presence of ozone.

Human skin lipids contain both saturated and unsaturated compounds; the latter react with ozone more rapidly.^20,21^ Among these, squalene is the most abundant compound in human skin lipids that readily reacts with ozone, followed by unsaturated fatty acids.^22−24^ Although extensive research has been dedicated to studying the gas-phase products resulting from the reaction of ozone with squalene and fatty acids,^22−33^ our understanding of particle formation by these reactions remains limited. Previous studies reported the formation of >10 nm secondary organic aerosols (SOAs) resulting from the reaction of ozone with surface-sorbed squalene.^34,35^ However, these studies have not delved into the NCA size range or explored the relative contribution of squalene and unsaturated fatty acids to NCA formation in the presence of ozone. In addition, our previous study revealed the dependence of NCA emissions on factors such as human age, clothing coverage, and air temperature and humidity but overlooked the potential influence of the ozone level.^17^

In addition, humans are a potent source of ammonia (NH_3_),^36^ which is known as an important player in atmospheric particle formation.^37−39^ It was previously presumed that human-emitted NH_3_ may play a role in NCA formation during the ozone–human reaction,^17^ a hypothesis that merits further investigation.

In view of these knowledge gaps, this study reports, for the first time, the formation of NCAs resulting from the squalene–ozone reaction. In a climate-controlled chamber, we measured NCAs generated from ozone’s reaction with surface-sorbed squalene and compared them with those resulting from the reaction of ozone with 6-hexadecenoic acid (C16:1n6), one of the most abundant unsaturated fatty acids in human skin lipids. We investigated the influence of ozone and NH_3_ levels on the generation of NCAs from squalene–ozone reactions. The results hold the potential to contribute to a deeper understanding of the mechanisms driving human-derived NCA generation and the effect of ozone chemistry on indoor NCA levels.

## Methods and Materials

### Experimental Procedure and Design

We performed experiments in a 1.9 m^3^ stainless-steel climate chamber (detailed in Section S1 and Figure S1). Reactants (squalene or C16:1n6) painted on a 0.24 m^2^ glass plate were exposed to ozone to investigate NCA formation. A typical human surface has an area of 1.8 m^2^. The reactive surface:volume ratio (0.24 m^2^/1.9 m^3^ = 0.13 m^–1^) corresponded to one person per 14 m^3^, which is commonly found indoors.^40^ A specific quantity of pure squalene (purity of >99%, Acros Organics, Thermo Fisher Scientific) or pure C16:1n6 (purity of >99%, Cayman) was dissolved in 10 mL of methanol, and then the mixture evenly painted on a glass plate using a glass stick. The painted glass plate was then placed on the stand inside the chamber. After the chamber door was closed, the chamber was ventilated with filtered air at an air change rate of 3 h^–1^ for 45 min to reduce the background contamination and then at a rate of 1 h^–1^ for an additional 45 min to stabilize the experimental conditions. Ozone was subsequently injected into the chamber to initiate the reaction at an air change rate of 1 h^–1^. After a reaction period of either 3 or 6 h, the glass plate was removed from the chamber and the measurement continued for an additional 20 min to capture the NCA decay. The full experimental procedure is visually detailed in Figure S3.

Table S1 lists the three experimental conditions investigated in this study. In experiments A comparing the reactions of ozone with squalene and C16:1n6, we applied 20 mg of squalene (equivalent to 83 mg/m^2^) and 5 mg of fatty acid (equivalent to 21 mg/m^2^) as reactants, respectively. The quantities were within the range detected on human skin. Each reaction lasted for 3 h with a steady-state ozone level of ∼60 ppb. In experiments B exploring the influence of the ozone level, 750 mg of squalene reacted with 90 ppb ozone for 3 h, followed by an additional 3 h period with 15 ppb ozone (in total 6 h). In experiments C, we studied a 3 h ozone reaction at 30 ppb without NH_3_, followed by an additional 3 h with ozone at 30 ppb and NH_3_ at 375 ppb, which approximates the NH_3_ levels inside the chamber occupied by an adult.^36^ In experiments B and C, the amount of squalene was increased to correspond to the typical total amount found in human skin lipids.

### Instrumentation and Quality Control

NCAs in the size range of 1.2–2.8 nm were sampled at a flow rate of 2.5 L/min and measured in real time at 2 min time intervals with a Nano Condensation Nucleus Counter (Airmodus A11 nCNC System, Airmodus). The principle of the measurement has been described in previous studies^17,41,42^ and in Section S2. The instrument was positioned immediately outside the chamber, and to minimize the particle sampling losses, we sampled NCAs with a core sampling probe at a carrier flow of 5 L/min.^43^

The ozone concentration inside the chamber was measured with a time resolution of 1 min with an ozone monitor (model 724, Tanabyte) at a sampling flow rate of 2.0 L/min. The level of NH_3_ was monitored at 30 s time intervals with a sampling flow rate of 140 mL/min using an NH_3_ analyzer (LSE NH_3_-1700, LSE Monitors). The air temperature and relative humidity (RH) were recorded using a HOBO UX90 sensor (Onset Inc.).

Before the experimental campaign, all instruments underwent full service and calibration. Due to limited resources, each experiment had one replicate, with variations typically falling within 20%, demonstrating the robust reproducibility of the results (Table S1).

### Data Analysis

Real-time NCA concentrations were derived through the inversion of raw data by the stepwise method^44^ using four size bins (1.2–1.5, 1.5–1.7, 1.7–1.9, and 1.9–2.8 nm). The average rate of emission of NCAs was obtained using the material-balance equation:
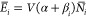
1where *N̅*_*i*_ (cm^–3^) is the steady-state particle number concentration for particle size *i*, *E̅*_*i*_ (particles per hour) is the particle number emission rate per hour for particle size *i*, *V* (*V* (cm^3^) is the chamber volume, α (h^-1^) is the air change rate, and β_*i*_ (h^−1^) is the particle deposition rate obtained via exponential fitting of the particle number concentration during the decay period after each experiment. Because of the relatively low particle concentration inside the chamber, we neglected the coagulation sink in the calculation.

## Results and Discussion

### Comparing the Reactions of Ozone with Squalene and Fatty Acid

**Figure 1:**
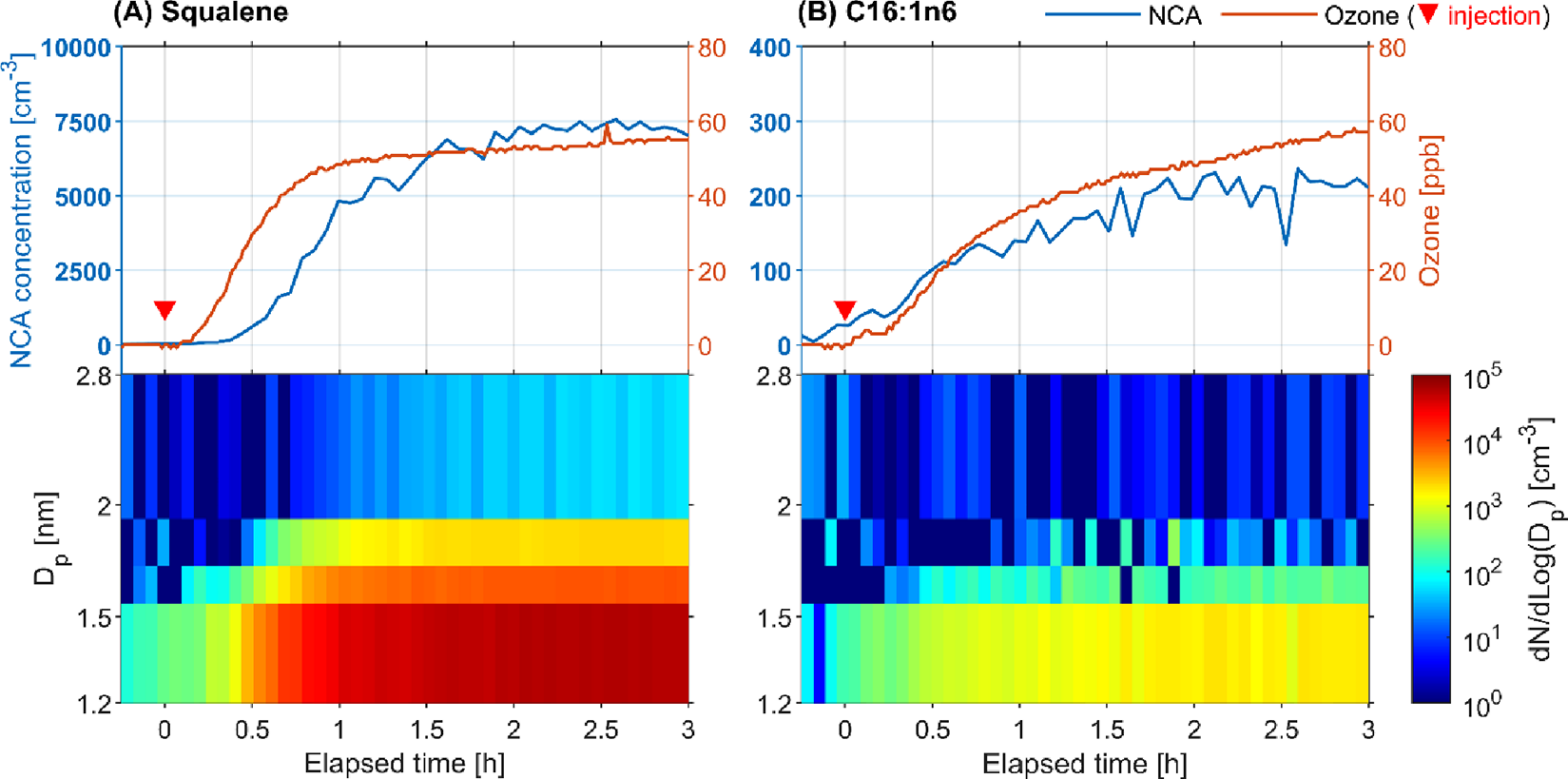
Time series of total NCA number concentration (1.2–2.8 nm) and ozone (top) and size-resolved NCA concentration (bottom) in experiments comparing the reactions of ozone with (A) squalene and (B) fatty acid C16:1n6. The upside-down triangle represents the moment at which ozone was injected into the chamber. *D*_p_ is the activation diameter determined by the nCNC instrument (detailed in Section S2). The data are from a single experiment from experiments A, whereas the replicate showed good reproducibility (Table S1 and Figure S4).

Figure 1 shows the time series of the ozone mixing ratio and NCA size distribution in experiments comparing the reactions of ozone with squalene and fatty acid C16:1n6. After ozone was injected into the chamber, the NCA levels in both reaction experiments started to increase. This finding supports the inference from our previous study that ozonolysis of human skin lipid compounds contributes to NCA formation.^17^ Although the steady-state ozone levels were similar in both experiments (55 and 56 ppb), the steady-state NCA levels were 40 times higher during ozonolysis of squalene relative to that of C16:1n6, indicating that squalene plays a dominant role in NCA formation when ozone reacts with human skin lipids. The size distributions of NCAs further demonstrated the disparity between the two reactions. The squalene–ozone reaction generated an abundant concentration of NCAs in the smallest size range (1.2–1.5 nm), which subsequently grew. In comparison, the ozonolysis of C16:1n6 emitted a much lower level of the smallest NCA, with no obvious signals detected for >1.7 nm NCAs (see also Table S1).

The reaction between ozone and C16:1n6 mainly produces gas-phase decanal and C_6_H_10_O_3_, with the latter potentially contributing to NCA formation via nucleation due to its low volatility.^22,23^ In comparison, the squalene–ozone reaction is notably more complex due to the presence of multiple unsaturated C=C bonds in squalene.^22,24,25^ In addition to generating gases such as acetone and 6-MHO,^22,23^ the squalene–ozone reaction can also produce low-volatility compounds that may nucleate, thereby forming NCAs. Squalene is mostly surface-sorbed indoors. Hence, the reaction of ozone with squalene vapors is negligible relative to surface chemistry.^22^ Previous studies examined surface-bound chemicals formed after the reaction of ozone with surface-sorbed pure squalene, including but not limited to 6-MHO, 4-OPA, geranylacetone, C17-trienal, secondary ozonides, and succinic acid.^25,45−49^ These low-volatility compounds are expected to contribute to NCA formation. In addition, Criegee intermediates formed during ozonolysis of squalene have the potential to propagate chain reactions in the autoxidation of unsaturated lipids, which is linked with NCA formation.^50,51^

### Influence of the Ozone Level

**Figure 2:**
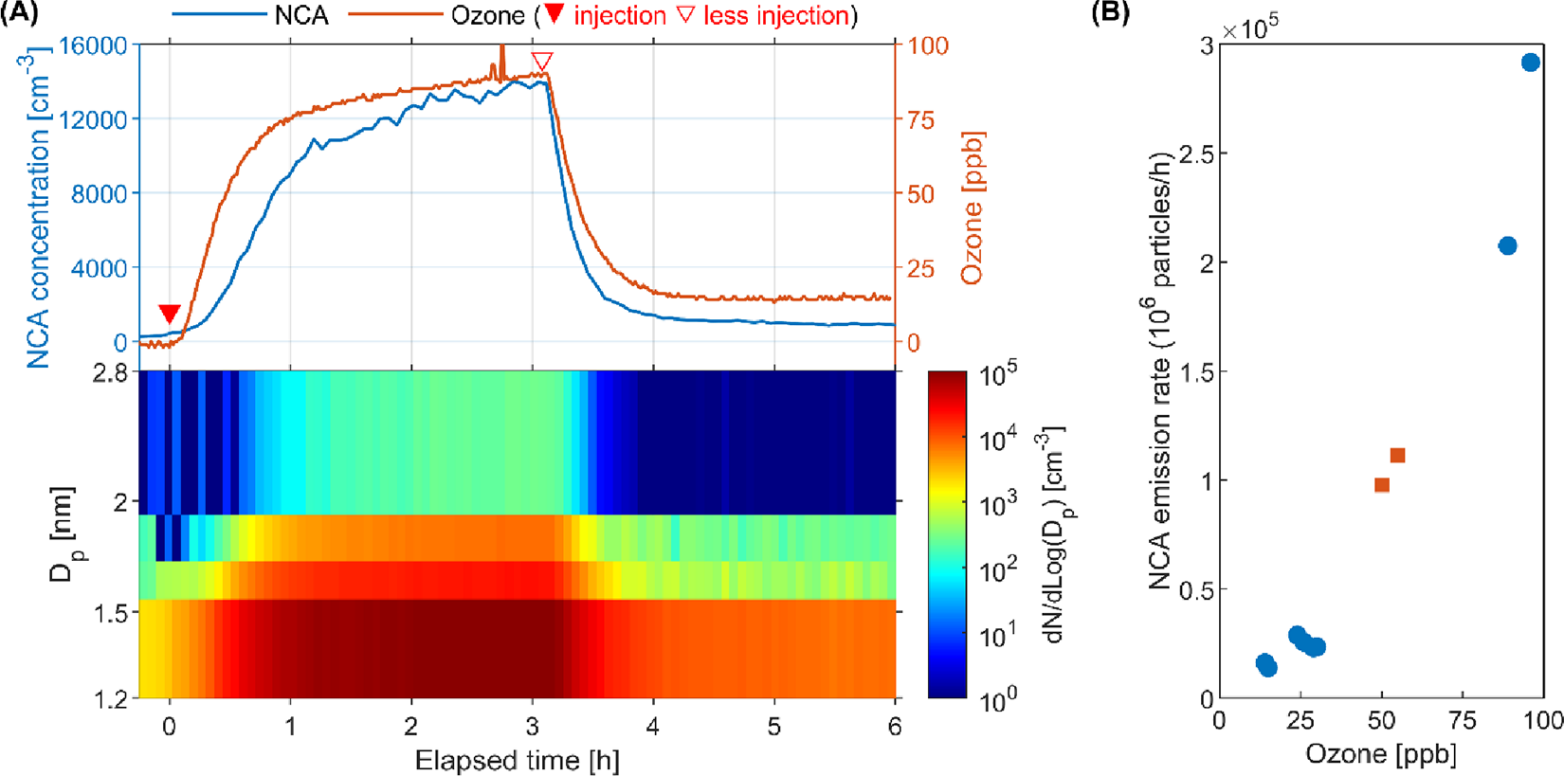
Influence of the ozone level on NCA emissions from ozone–squalene chemistry. (A) Time-series plot of the NCA concentration and size distribution following a decrease in the level of ozone. The upside-down filled triangle represents the moment at which ozone was injected into the chamber at a high flow rate (target at 90 ppb), whereas the upside-down hollow triangle represents the moment at which less ozone was injected (target at 15 ppb). The data are from a single experiment from experiments B, whereas the replicate showed good reproducibility (Table S1 and Figure S4). (B) Correlation between the steady-state ozone level and NCA emission rates across all experimental runs for the reaction of ozone with squalene. The two square dots represent experiments using 20 mg of squalene, whereas the round dots represent experiments using 750 mg of squalene.

The NCAs generated by the squalene–ozone reaction were strongly influenced by the ozone level, as illustrated in Figure 2A. The NCA level inside the chamber reached 1.4 × 10^4^ cm^–3^ at a steady-state ozone level of 89 ppb. When less ozone was injected, causing a decrease in ozone levels, the NCA concentration followed the trend of ozone until reaching a new steady-state level of 1.0 × 10^3^ cm^–3^ when the ozone concentration stabilized at 14 ppb. Such an obvious decrease was observed for all size bins, especially for >1.7 nm particles. Combining ozone and NCA emission rate data from all squalene–ozone experiments revealed a positive correlation between them [*N* = 10; *R*^2^ = 0.95 (Figure 2B)]. Similar correlations were observed between ozone and >10 nm SOA for the ozonolysis of squalene,^34,35^ skin-oiled clothing,^52^ and occupants.^53^ It is noteworthy that this correlation held true despite the use of different amounts of squalene applied to the glass plate [20 and 750 mg (Figure 2B)]. This finding indicates that squalene was in excess during the squalene–ozone reaction,^54^ which was mainly constrained by the gas-phase ozone level and transportation, aligning with the observed ozone–human reaction.^17^ The results also highlighted the importance of controlling the indoor ozone level in reducing the level of exposure of humans to NCAs and other products initiated from indoor ozone chemistry.

The absence of data on ozone loss impedes our ability to calculate the NCA yield. Our recent investigation of the impact of air change rates on NCA formation via ozone–human chemistry indicated a positive correlation between the NCA formation rate and the ozone removal rate, which provided insights into this study regarding the dependence of NCA generation on ozone level.^19^

### Influence of the NH_3_ Level

**Figure 3:**
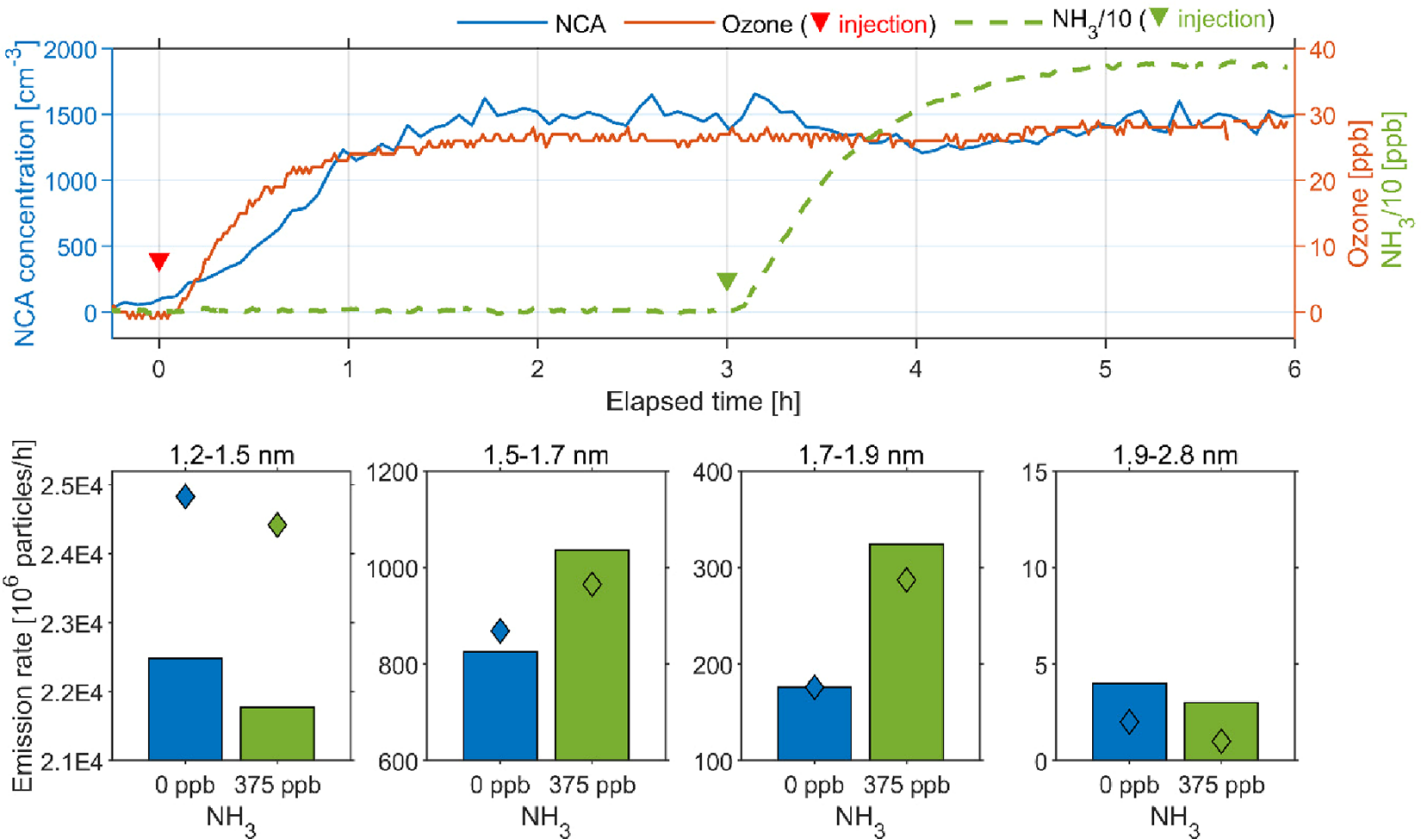
Influence of NH_3_ level on NCA emissions from ozone–squalene chemistry. The top plot shows time-series concentrations of NCAs, ozone, and NH_3_ inside the chamber. The upside-down red triangle represents the moment at which ozone was injected into the chamber (target at 30 ppb), whereas the upside-down green triangle represents the moment at which NH_3_ was injected (target at 375 ppb). Note that the NH_3_ concentrations were divided by 10. The bottom charts represent the dependence of steady-state NCA emission rates for each size bin on the NH_3_ levels. The bar plots are from a single experiment from experiments C, whereas the diamond dots represent the replicate, indicating good reproducibility (also in Table S1).

The influence of NH_3_ on NCA formation was less pronounced compared to the influence of ozone, as shown in Figure 3. Upon injection of NH_3_ into the chamber, there was a slight decrease in NCA level, although the ozone concentration remained the same. The decrease was mainly caused by the decrease in the level of the smallest NCAs (1.2–1.5 nm). However, emissions of larger NCAs (1.5–1.9 nm) increased with the NH_3_ level, especially for 1.7–1.9 nm NCAs, with the emission rate doubled at 375 ppb NH_3_ (Figure 3 and Table S1). This indicates that NH_3_ could contribute to the stabilization and growth of NCAs. NH_3_ has been found to enhance SOA formation during ozone–terpene reactions.^55,56^ In addition, NH_3_ can react with acidic products from squalene–ozone reaction to form salts.^39,57^ When such a reaction happens between gas-phase NH_3_ and freshly formed particle-phase acidic products, it may lead to a slight decrease in the level of the smallest NCAs but subsequently promote their growth.^58^

### Limitations and Future Outlook

We selected C16:1n6 as the representative unsaturated fatty acid owing to its relatively high abundance in human skin lipids.^23,59^ Other fatty acids and other unsaturated skin lipid compounds (such as esters and waxes) may have different NCA generation behaviors when they react with ozone. However, given the complexity and diversity of squalene–ozone reaction processes, we suspect that squalene is still the key contributor to NCA generation via ozone chemistry on human surfaces. Moreover, the ozonolysis of squalene may vary when occurring on real human skin relative to a glass plate. This process could be influenced by the skin temperature, moisture content, surface roughness, and clothing coverage.

In addition, this study examined the ozonolysis of squalene and fatty acid separately. The reaction of ozone with the mixture of these compounds, as they exist in real human skin lipids, may synergistically enhance NCA generation. This is expected due to the introduction of more low-volatility products and the potential reactions among these products, which merit additional examination.

It is worth noting that the smallest size bin of the detected NCAs could consist of large organic gaseous clusters formed during the ozonolysis of squalene. After the installation of a zero-check HEPA filter at the inlet of the nCNC during the squalene–ozone reaction, the instrument showed <10 particles/cm^3^ in the smallest size bin, whereas there were no signals for larger NCAs (Figure S5). Although the HEPA filter may also eliminate low-volatility gases to some extent, the efficiency is considerably lower than the efficiency of filtering aerosol particles.^60^ Hence, it indicates that the detected NCAs during the ozonolysis of squalene in this study were predominantly in the aerosol phase. Nevertheless, it should be noted that the activation diameters of the detected NCAs, presumably organic, can differ from those of the calibration compounds (monodisperse NiCr oxide particles), as suggested by Rörup et al.^61^ The exact size of NCAs formed by ozonolysis of squalene merits further investigation.

In addition to the ozone and NH_3_ levels investigated in this study, another potential influencing factor that merits further study is RH. RH can alter the gaseous products from the ozonolysis of squalene as well as the gas-to-particle conversion process,^24,34,62^ as the water molecules may help to stabilize the initial clusters. Our previous studies showed inconsistent results with respect to the influence of RH on NCAs formed by ozone–human/clothing chemistry. In ozone–human experiments, increased RH from 18% to 35% enhanced NCA generation,^17^ whereas the level of NCAs decreased when RH increased from 40% to 65% during ozonolysis of skin-oiled clothing.^18^ Hence, future studies should examine the effect of RH on gaseous- and particle-phase products derived from ozone–squalene chemistry.

The NCA emission rates obtained from the squalene–ozone reaction in this study (Table S1) were on the same order of magnitude as those observed for the ozone–human reaction in another chamber study,^17^ which were 2–3 orders of magnitude lower than emissions from cooking^11^ and the use of three-dimensional (3D) printers.^15^ However, sources such as cooking and 3D printing are sporadic, whereas the squalene–ozone reaction takes place continuously whenever humans encounter ozone and thus contributes substantially to daily indoor NCA levels (discussed in Section S3). Moreover, we expect that NCAs generated from ozone–squalene reactions would be more pronouncedly distributed close to the reactive surface relative to the bulk air.^63^ This may imply that the NCA level can be higher in the peri-human microenvironment than in room air (the phenomenon termed “personal cloud”^64−66^). Such a spatial variation of NCAs generated from ozone–squalene chemistry merits future examination.

This study is the first to report NCA formation from the squalene–ozone reaction. This experimentally confirms that NCAs appear to be formed from ozone–squalene reactions in ozone–human chemistry and enhances our understanding of the role of ozone and NH_3_ in the process. The interpretation can be further enhanced by analyzing gas-phase product measurements in parallel, which should be performed in future studies. In addition, analysis of the chemical composition of the formed particles has the potential to reveal the oxidation products or water-bound molecules that contribute to NCA generation. Finally, the health effect of the generated NCAs remains to be further explored.

## Supporting Information Available

Climate chamber (Section S1), principle of NCA measurements (Section S2), contribution to daily indoor NCA levels (Section S3), schematic of the climate chamber setup (Figure S1) RH levels in experiments (Figure S2), visualization of the experimental procedures (Figure S3), NCA levels in experiments (Figure S4), example of a zero check of the instrument (Figure S5), and summary of the experimental conditions and associated NCA emission rates (Table S1) (PDF)
