# Supplementary material for: Nanocluster Aerosols from Ozone–Human Chemistry Are Dominated by Squalene–Ozone Reactions
Source: Environ Sci Technol Lett. 2024 Jun 21;11(7):716–22. doi: 10.1021/acs.estlett.4c00289 (PMC11238579; doi:10.1021/acs.estlett.4c00289)
Supplement: Supplementary file 1 — ez4c00289_si_001.pdf [file ez4c00289_si_001.pdf]

Supporting Information for

Nanocluster Aerosols from Ozone-Human Chemistry Are Dominated by Squalene-Ozone Reactions

Shen Yang, * Dusan Licina

Human-Oriented Built Environment Lab, School of Architecture, Civil and Environmental Engineering, École Polytechnique Fédérale de Lausanne (EPFL), 1015 Lausanne, Switzerland

Corresponding authors:

Shen Yang (shen.yang@epfl.ch)

This supporting file includes:

Section S1. Climate chamber

Section S2. Principle of NCA measurements

Section S3. Contribution to daily indoor NCA levels

Fig. S1. Schematic figure of the climate chamber setup

Fig. S2. RH levels in experiments

Fig. S3. Visualization of experiment procedures

Fig. S4. NCA levels in experiments

Fig. S5. An example of zero check of the instrument

Table S1. Summary of experimental conditions and associated NCA emission rates

Section S1. Climate chamber

The chamber was ventilated by filtered compressed air to ensure low background levels of particles (<100 #/cm^3 below 10 nm) and ozone (<1 ppb). The air was supplied through two side inlets and exhausted via two side outlets. The chamber air temperature was controlled at 24 °C by water bath controllers, while the relative humidity was maintained at 40 ± 5% by adjusting airflow passing through the gas wash bottle (Fig. S2). Ozone was generated by a Jelight 600 UV generator (Jelight Co. Inc., USA). In experiments with NH_3, we injected NH_3 from a gas cylinder (10 ppm, purity >99.9%, Cabagas Inc., CH). A stainless-steel stand inside the chamber held the glass plate for conducting ozone-squalene and -fatty acid reactions. Two desk fans facing the chamber walls ensured air mixing. All surfaces were thoroughly cleaned by methanol and then distilled water prior to each experiment.

Section S2. Principle of NCA measurements

NCA levels within the chamber were measured using a Nano Condensation Nucleus Counter (Airmodus A11 nCNC System, Airmodus, Finland), which incorporates a Particle Size Magnifier (PSM A10) and a Condensation Particle Counter (CPC A20). The PSM serves to enlarge small particles to a size detectable by the CPC, through a mixing-type mechanism.[1] This mixing ratio can be swiftly adjusted, resulting in varied smallest particles that can be amplified by the PSM. Continuous scanning of the mixing ratio enables the measurement of aerosol size distribution within the 1-4 nm range.

The PSM operated in scanning mode with the saturator flow scanning consistently at a constant rate, enabling the detection of aerosols (named activation size) ranging from 1.2 to 4 nm. A complete scan comprised two 2-minute intervals: first, the saturator flow increased from 0.1 to 1.3 L/min (up-scan), followed by a return to 0.1 L/min (down-scan). Typically, aerosol particle concentrations for each size bin were averaged over the two periods, resulting in a time resolution of 4 minutes. In instances where NCA concentrations exhibited substantial variations, a 2-minute resolution was employed to better capture the NCA dynamics.

Compared to alternative particle detection technologies like the Scanning Mobility Particle Sizer (SMPS), this method minimizes losses of the smallest aerosols as it does not involve prior size selection or particle charging. This instrument has found extensive use in measuring atmospheric outdoor NCA[2] but its utilization indoors has been relatively limited.[3–5]

Section S3. Contribution to daily indoor NCA levels

The primary goal of this study was to explore which chemical process predominantly generates NCA in ozone-human chemistry. Instead of attempting to fully replicate realistic indoor environment conditions, this study serves as a crucial stepping stone, providing insights that will inform and guide future research conducted in real indoor environments. However, it is noteworthy that several parameters in the experimental climate chamber closely mirrored typical indoor environments, such as temperature and humidity (24 °C and 40%, respectively),[6] air change rate (1 h^{-1}),[7] and ozone level (15-96 ppb). [8–10] Furthermore, NCA deposition rates (7.0-7.9 h^{-1}) inside the chamber closely approximated measurements or models observed in indoor environments.[11] The applied squalene quantities (21-83 mg/m^2) were within the range typically detected on human skin. [12–15] Hence, based on the emission rates obtained from this study, we can expect a similar NCA level (10^3-10^4 particles/cm^3) in typical indoor environments generated via ozone-human chemistry. Such a level is at the same order of magnitude of the limited measurements of indoor NCA concentrations without other prominent and temporary sources,[16] such as candle burning, cooking, and mopping.[3] It is worth mentioning that the results in this study were obtained with the air mixing fans activated. Recent study has shown that the operation of mixing fans substantially decreases NCA formation and subsequent growth.[17] Therefore, in daily indoor environments without fan operations, we can expect a larger contribution of ozone-human chemistry to indoor NCA and ultrafine particle levels. On the other hand, our previous study demonstrated that increasing clothing coverage led to decreased NCA emissions.[5] Hence, while presence of occupants' clothing is expected to mitigate the NCA levels, we anticipate a significant net increase in NCA levels in the absence of mixing fans. The implication is that ozone-human chemistry considerably contributes to daily build-up of indoor NCA levels. However, given the sensitivity and complexity of the gas-to-particle conversion processes and NCA measurements, [18,19] as well as the potential effects on NCA formation caused by coagulation and/or condensation onto existing larger particles indoors and indoor air movement, future investigations into squalene-ozone reaction in various indoor environments, including off-body squalene ozonolysis, are warranted. [20]

In addition to indoor environments, ozone-human chemistry may also contribute to outdoor NCA. However, it should be noted that the emission rates obtained in this study cannot be directly applied to outdoors, as the outdoor environment significantly differs from indoors, particularly in air movement and surface-to-volume ratio, which are key factors influencing the

dynamics and fate of NCA.[17] Future outdoor field measurements can address the potential contribution of ozone-human chemistry to the atmospheric NCA.

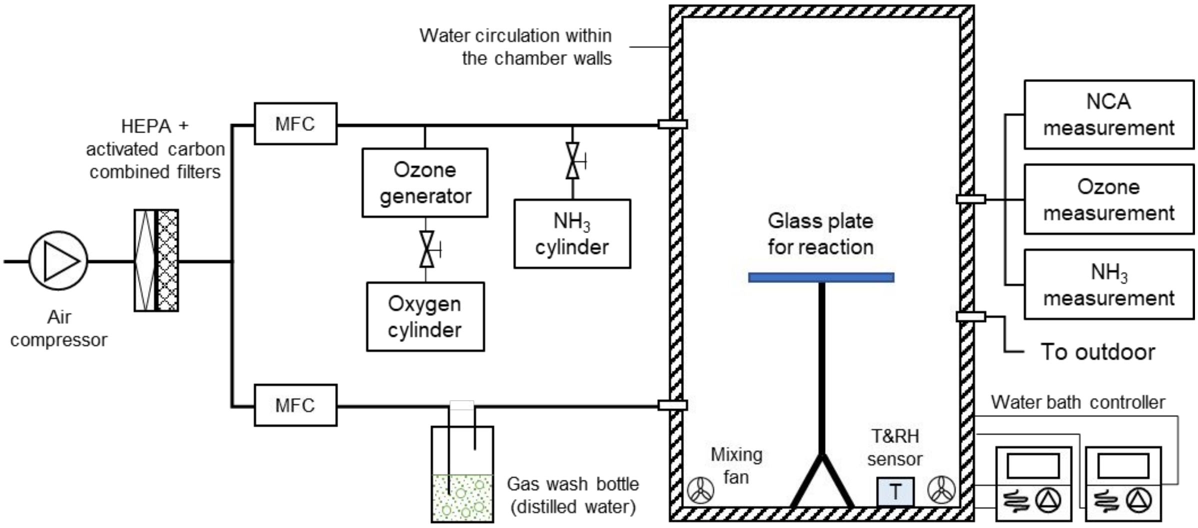

Fig. S1. Schematic figure of the climate chamber setup. The chamber air temperature was controlled by the temperature of the chamber surfaces though which water was circulating and whose temperature was controlled by two water bath controllers. The relative humidity was controlled by adjusting airflow passing the gas wash bottle using mass flow controllers (MFC).

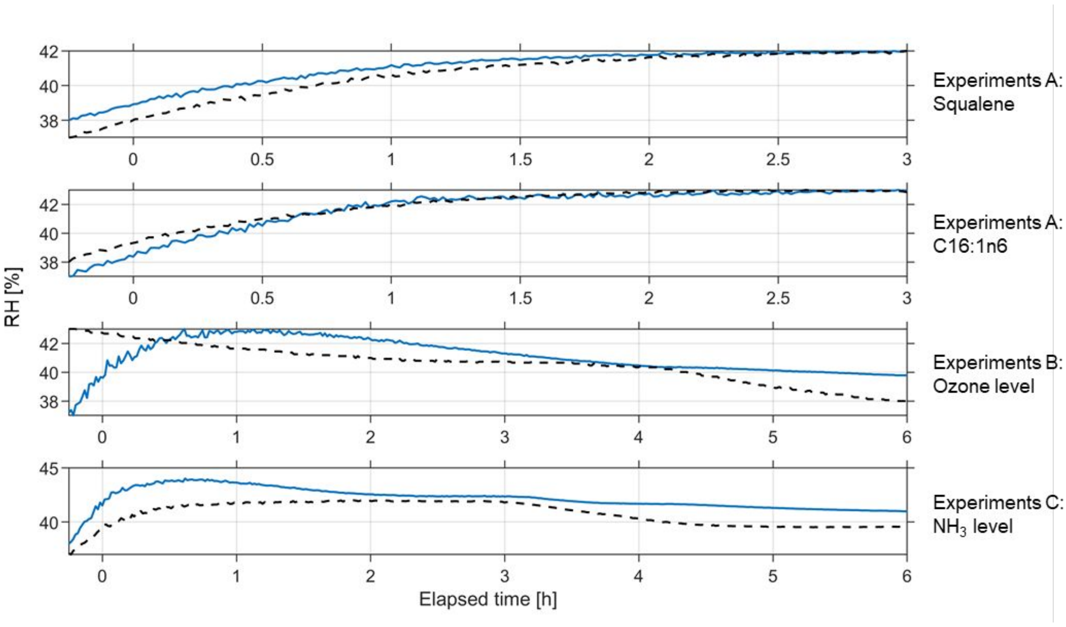

Fig. S2. Time-series plots of RH level in each experimental run, controlled at 40±5%. The dashed lines represent results from replicate experiments.

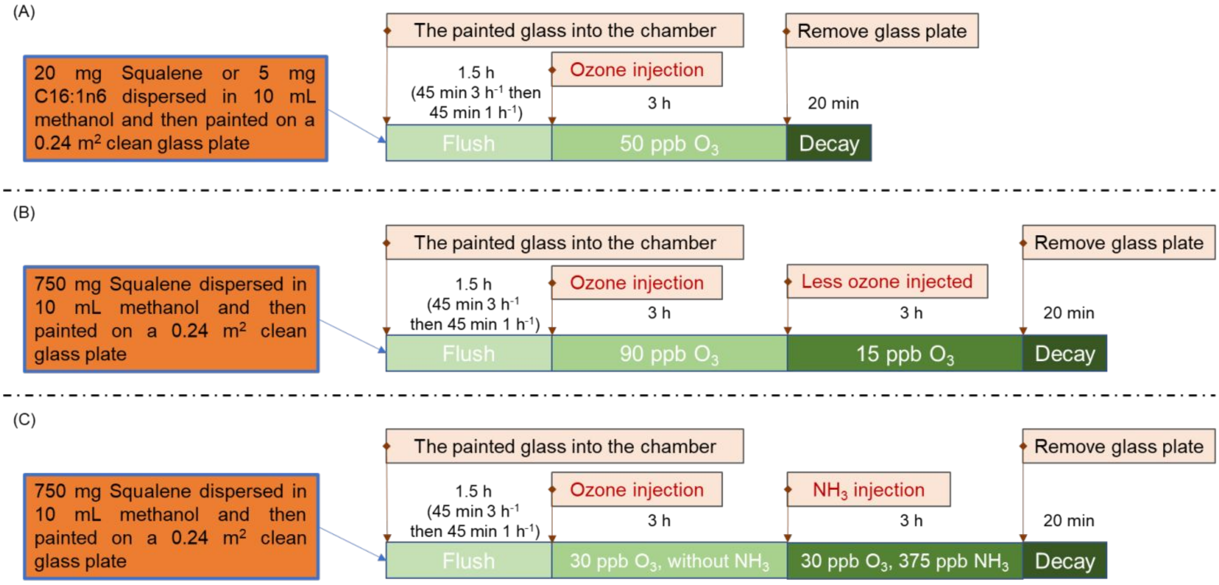

Fig. S3. Experiment procedures for (A) comparing ozone reaction with squalene and C16:1n6; (B) investigating the impact of ozone level; and (C) investigating the impact of NH_3 level, where ozone was injected throughout the 6 h reaction.

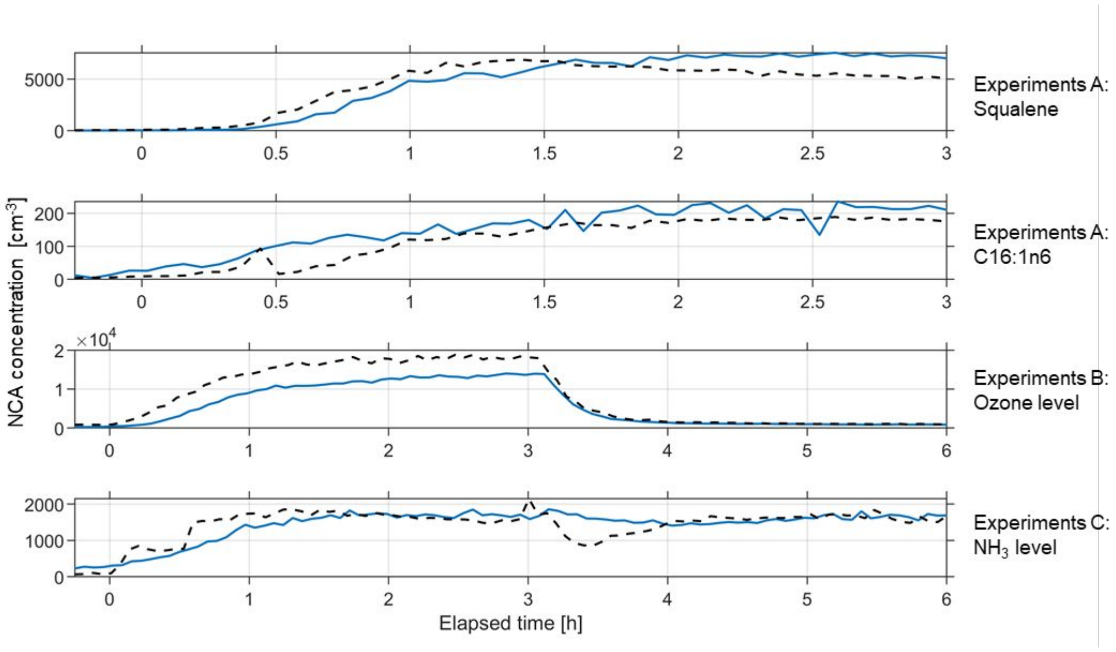

Fig. S4. Time-series plots of NCA concentration in each experimental run. The dashed lines represent results from replicate experiments.

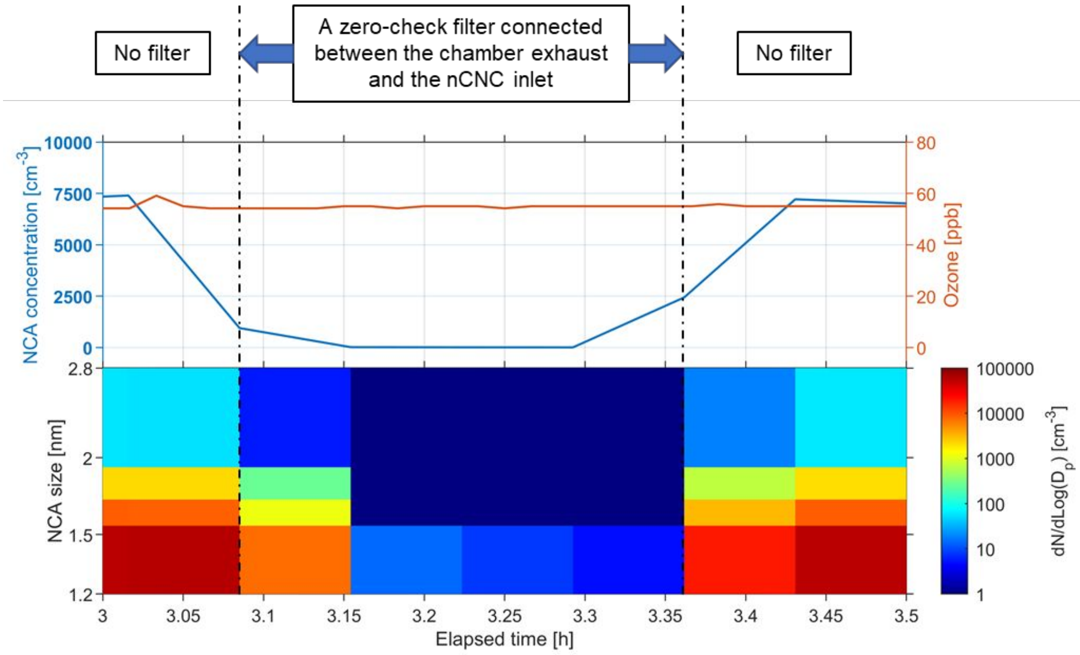

Fig. S5. An example of zero-check of the nCNC instrument during the ozone-squalene reaction. This zero check was performed during one test in Experiments A for squalene ozonolysis. A zero-check filter was connected between the chamber exhaust and the nCNC inlet after 3-h reaction. As seen, during the zero-check, the instrument background showed <10 particles/cm^3, whereas the measured NCA went back to the previous level after the filter being disconnected. It indicates the low background of the instrument itself, and proves that the measured clusters were predominantly in particle phase.

Table S1. Summary of experimental conditions and associated nanocluster aerosol (NCA) emission rates. SS: steady state, Rep.: replicate experiment

				Emission rate (10^6 particles/h)				
Exp. Set	Reactant	Ozone SS	NH_3 SS	1.9-2.8 nm	1.7-1.9 nm	1.5-1.7 nm	1.2-1.5 nm	Total NCA
A. Comparing squalene and fatty acid	20 mg squalene	50	5	56	1075	4907	91611	97649
	20 mg squalene (Rep.)	55	4	137	1861	6374	103033	111404
	5 mg fatty acid	51	4	0	17	101	3065	3183
	5 mg fatty acid (Rep.)	56	7	17	14	172	2975	3178
B. Influence of ozone	750 mg squalene (the 1st 3 h)	89	3	652	6531	15278	185022	207484
	750 mg squalene (the 2nd 3 h)	15	2	1	222	329	13361	13913
	750 mg squalene (the 1st 3 h, Rep.)	96	2	1233	10372	22275	257688	291569
	750 mg squalene (the 2nd 3 h, Rep.)	14	10	1	164	299	15767	16231
C. Influence of NH_3	750 mg squalene (the 1st 3 h)	30	0	4	176	826	22479	23485
	750 mg squalene (the 2nd 3 h)	29	375	3	324	1036	21774	23136
	750 mg squalene (the 1st 3 h, Rep.)	24	4	2	176	869	24827	25875
	750 mg squalene (the 2nd 3 h, Rep.)	26	380	1	287	965	24415	25667

References:

(1) Vanhanen, J.; Mikkilä, J.; Lehtipalo, K.; Sipilä, M.; Manninen, H. E.; Siivola, E.; Petäjä, T.; Kulmala, M. Particle Size Magnifier for Nano-CN Detection. *Aerosol Science and Technology* 2011, *45* (4), 533–542. https://doi.org/10.1080/02786826.2010.547889.

(2) Kontkanen, J.; Lehtipalo, K.; Ahonen, L.; Kangasluoma, J.; Manninen, H. E.; Hakala, J.; Rose, C.; Sellegri, K.; Xiao, S.; Wang, L.; Qi, X.; Nie, W.; Ding, A.; Yu, H.; Lee, S.; Kerminen, V.-M.; Petäjä, T.; Kulmala, M. Measurements of Sub-3 Nm Particles Using a Particle Size Magnifier in Different Environments: From Clean Mountain Top to Polluted Megacities. *Atmos Chem Phys* 2017, *17* (3), 2163–2187. https://doi.org/10.5194/acp-17-2163-2017.

(3) Patel, S.; Sankhyan, S.; Boedicker, E. K.; DeCarlo, P. F.; Farmer, D. K.; Goldstein, A. H.; Katz, E. F.; Nazaroff, W. W.; Tian, Y.; Vanhanen, J.; Vance, M. E. Indoor Particulate Matter during HOMEChem: Concentrations, Size Distributions, and Exposures. *Environ Sci Technol* 2020, *54* (12), 7107–7116. https://doi.org/10.1021/acs.est.0c00740.

(4) Rosales, C. M. F.; Jiang, J.; Lahib, A.; Bottorff, B. P.; Reidy, E. K.; Kumar, V.; Tasoglou, A.; Huber, H.; Dusanter, S.; Tomas, A.; Boor, B. E.; Stevens, P. S. Chemistry and Human Exposure Implications of Secondary Organic Aerosol Production from Indoor Terpene Ozonolysis. *Sci Adv* 2022, *8* (8). https://doi.org/10.1126/sciadv.abj9156.

(5) Yang, S.; Licina, D.; Weschler, C. J.; Wang, N.; Zannoni, N.; Li, M.; Vanhanen, J.; Langer, S.; Wargocki, P.; Williams, J.; Bekö, G. Ozone Initiates Human-Derived Emission of Nanocluster Aerosols. *Environ Sci Technol* 2021, *55* (21), 14536–14545.

(6) Földváry Ličina, V.; Cheung, T.; Zhang, H.; de Dear, R.; Parkinson, T.; Arens, E.; Chun, C.; Schiavon, S.; Luo, M.; Brager, G.; Li, P.; Kaam, S.; Adebamowo, M. A.; Andamon, M. M.; Babich, F.; Bouden, C.; Bukovianska, H.; Candido, C.; Cao, B.; Carlucci, S.; et al. Development of the ASHRAE Global Thermal Comfort Database II. *Build Environ* 2018, *142*, 502–512. https://doi.org/10.1016/j.buildenv.2018.06.022.

(7) Howard-Reed, C.; Wallace, L. A.; Ott, W. R. The Effect of Opening Windows on Air Change Rates in Two Homes. *J Air Waste Manage Assoc* 2002, *52* (2), 147–159. https://doi.org/10.1080/10473289.2002.10470775.

(8) Nazaroff, W. W.; Weschler, C. J. Indoor Ozone: Concentrations and Influencing Factors. *Indoor Air* 2022, *32* (1). https://doi.org/10.1111/ina.12942.

(9) Salonen, H.; Salthammer, T.; Morawska, L. Human Exposure to Ozone in School and Office Indoor Environments. *Environ Int* 2018, *119*, 503–514. https://doi.org/10.1016/j.envint.2018.07.012.

(10) Bhangar, S.; Nazaroff, W. W. Atmospheric Ozone Levels Encountered by Commercial Aircraft on Transatlantic Routes. *Environmental Research Letters* 2013, *8* (1), 014006. https://doi.org/10.1088/1748-9326/8/1/014006.

(11) Wallace, L.; Jeong, S.; Rim, D. Dynamic Behavior of Indoor Ultrafine Particles (2.3-64 Nm) Due to Burning Candles in a Residence. *Indoor Air* 2019, *29* (6), 1018–1027. https://doi.org/10.1111/ina.12592.

(12) Yao, M.; Zhao, B. Ozone Reactive Compounds Measured in Skin Wipes from Chinese Volunteers. *Build Environ* 2021, *188*, 107515. https://doi.org/10.1016/J.BUILDENV.2020.107515.

(13) Downing, D. T.; Strauss, J. S. Synthesis and Composition of Surface Lipids of Human Skin. *Journal Of Investigative Dermatology* 1974, *62* (3), 228–244. https://doi.org/10.1111/1523-1747.ep12676793.

(14) Downing, D. T.; Strauss, J. S.; Pochi, P. E. Variability in the Chemical Composition of Human Skin Surface Lipids. *J Invest Dermatol* 1969, *53* (5), 322–327. https://doi.org/10.1038/jid.1969.157.

(15) Ramasastry., P.; Downing., D. T.; Pochi., P. E.; Strauss., J. S. Chemical Composition of Human Skin Surface Lipids from Birth to Puberty. *J Invest Dermatol* 1970, *54* (2), 139–144.

(16) Amouei Torkmahalleh, M.; Turganova, K.; Zhigulina, Z.; Madiyarova, T.; Adotey, E. K.; Malekipirbazari, M.; Buonanno, G.; Stabile, L. Formation of Cluster Mode Particles (1–3 Nm) in Preschools. *Science of The Total Environment* 2022, *818*, 151756. https://doi.org/10.1016/j.scitotenv.2021.151756.

(17) Yang, S.; Müller, T.; Wang, N.; Bekö, G.; Zhang, M.; Merizak, M.; Wargocki, P.; Williams, J.; Licina, D. Influence of Ventilation on Formation and Growth of 1–20 Nm Particles via Ozone–Human Chemistry. *Environ Sci Technol* 2024, *58* (10), 4704–4715. https://doi.org/10.1021/acs.est.3c08466.

(18) Kroll, J. H.; Seinfeld, J. H. Chemistry of Secondary Organic Aerosol: Formation and Evolution of Low-Volatility Organics in the Atmosphere. *Atmos Environ* 2008, *42* (16), 3593–3624. https://doi.org/10.1016/j.atmosenv.2008.01.003.

(19) Kangasluoma, J.; Cai, R.; Jiang, J.; Deng, C.; Stolzenburg, D.; Ahonen, L. R.; Chan, T.; Fu, Y.; Kim, C.; Laurila, T. M.; Zhou, Y.; Dada, L.; Sulo, J.; Flagan, R. C.; Kulmala, M.; Petäjä, T.; Lehtipalo, K. Overview

of Measurements and Current Instrumentation for 1–10 Nm Aerosol Particle Number Size Distributions. *J Aerosol Sci* 2020, *148*, 105584. https://doi.org/10.1016/j.jaerosci.2020.105584.
(20) Zhang, M.; Gao, Y.; Xiong, J. Characterization of the Off-Body Squalene Ozonolysis on Indoor Surfaces. *Chemosphere* 2022, *291*, 132772. https://doi.org/10.1016/j.chemosphere.2021.132772.
